# Desires and Attitudes towards Telepharmacy Medicine Delivery

**DOI:** 10.3390/ijerph192013571

**Published:** 2022-10-20

**Authors:** Konkanok Wattana, Siranee Yongpraderm, Tida Sottiyotin, Najmee Adulyarat, Cheewarat Suntonchainugul, Natcha Chinakarapong, Thanutcha Suwanchatre

**Affiliations:** 1 Department of Pharmaceutical Care, Walailak University, Tha Sala, Nakhon Si Thammarat 80160, Thailand; 2 Drug and Cosmetics Excellence Center, Walailak University, Tha Sala, Nakhon Si Thammarat 80161, Thailand

**Keywords:** attitude, desire, medicine delivery, telepharmacy, service

## Abstract

The COVID-19 pandemic has limited healthcare services for patients with non-communicable diseases (NCDs). Hospital pharmacy departments in Thailand apply a new normal pharmacy service known as “telepharmacy” to serve remote pharmacy practice and deliver medication to patients. Current knowledge clearly shows the benefit of each medicine delivery method, but the study of patient’s desires and attitudes towards all drug delivery methods is still limited. To fill the gap, this study aims to investigate desires and attitudes about drug delivery methods among Thai patients living with NCDs who need regular and continuous care. The sample was included by accidentally randomized technique at NCD clinics of the southern Thailand tertiary care hospital. Data were collected between January and March 2021 by a questionnaire that contained three sections: the currently received medicine delivery method, the desires and attitudes about the medicine delivery system, and patients’ demographic information. The majority of patients were women aged 60 years who earned less than 10,000 THB (263.85 USD), were enrolled in the Civil Servant Medical Benefit Scheme (CSMBS), lived 0–15 km from the hospital, living with hypertension, had 1–4 prescribed medications, visited the doctor every 3 months, and received the conventional drug delivery method. The result showed that only the subdistrict health promotion hospital (SHPH) medicine delivery method was at a high level of desire, while the rest including conventional, drug store, postal pharmacy, and drive-thru medicine delivery methods were at medium. Attitudes toward the quality of medicine delivery methods consisted of five dimensions: confidence, timeliness, reliability, empathy, and facilities. Thai NCD patients had positive attitudes toward SHPH and drug store medicine delivery methods that could be seen from the high level of attitude score across all dimensions, while postal pharmacy and drive-thru delivery methods received medium-level attitude scores across all five dimensions.

## 1. Introduction

Non-communicable diseases (NCDs) are chronic diseases caused by various variables that include behavior, physiology, genetics, and the environment. NCDs affect all age groups, particularly the elderly. The primary types of NCDs are cardiovascular diseases, chronic respiratory diseases and diabetes [1]. In 2020, the Division of Non-Communicable Diseases of Thailand reported a higher mortality rate from four NCD types than in previous years [2]. Discontinuous medication is one factor that affects hospital admissions and mortality [3,4,5]. The COVID-19 pandemic resulted in social distancing and transportation restrictions, which impacted patients’ obtainment of hospital services and led to discontinuous drug treatment. Consequently, the pharmacy unit needed to serve a new normal service known as “telepharmacy” which involved both practices of remote pharmacy, such as patient counseling via phone; drug information provision via phone message; drug dispensing via face time, and various methods of medicine delivery to the patients [6].

Nowadays, Thailand’s healthcare service has five methods for delivering medicines, including the conventional drug delivery method whereby patients receive their medicines at a hospital pharmacy department; subdistrict health promotion hospital (SHPH) drug delivery method whereby patients receive their medicines at a Thai primary care service unit, namely subdistrict (Tambon) health promotion hospital; drug store delivery method whereby patients receive their medicines at community pharmacies or drug stores near their homes; the postal pharmacies method whereby patients receive their medicines via post; and the drive-thru method whereby patients receive their medicines by drive-thru service at a hospital. The benefits of each medicine delivery method are clearly presented; for example, in Spain, NCD patients are satisfied with the drugs received from community pharmacies and willing to continuously use this service [7]. In South Africa, as measured by questionnaires that compare patient satisfaction with the dispensing processes of community pharmacies and postal pharmacies, the overall and technical satisfaction levels do not differ considerably between the two groups. The postal group reported significant differences in financial satisfaction, while community pharmacy users reported higher satisfaction scores in counseling or explanations [8]. Additionally, patients in a public hospital chose to have their medications delivered to their homes. Factors such as age, employment status, distance from the home to the clinic, frequency of medicine refills, and pharmacy waiting time impacted their involvement in the medicine delivery service. Those with residences within 1 km of the clinic, and who wait more than 6 h at the pharmacy are associated with the demand for pharmaceutical delivery services. Once-a-month refills were strongly connected with a lower number of patients requiring the drug delivery service, but refills were significantly associated with high demand every 2 months [9]. Furthermore, in Thailand, previous research revealed that telepharmacy with home drug delivery decreased drug-related problems and increased patient satisfaction [10].

However, study of clients’ desires and attitudes towards all drug delivery methods is still limited. To fill the gap in knowledge, this study aimed to investigate desires and attitudes regarding five drug delivery methods among Thai patients living with NCDs who need regular and continuous care. The information may be beneficial for policy decisions on drug delivery routes that meet the demands of NCD patients.

## 2. Materials and Methods

### 2.1. Study Design

A descriptive, cross-sectional study was conducted to assess patients’ desires and attitudes toward telepharmacy, namely, medicine delivery services, in a tertiary hospital in southern Thailand.

### 2.2. Participants and Context

An NCD clinic in a Southern Thailand tertiary care hospital was purposively select-ed as a research setting. Patients with comorbidity diseases including hypertension, diabetes mellitus, asthma, cardiovascular disease, bone and joint disease, gastrointestinal and hepatic disease, skin disease, otorhinolaryngologic disease, genitourinary disease, and kidney disease were accidentally randomized and selected. The total sample size of 406 was determined using Cochran’s formulation. This study only included patients who were at least 18 years of age, were literate, and consented to participate in the study. The inability to finish the questionnaire resulted in exclusion.

### 2.3. Data Collection

Following ethical approval (6 January 2021), the questionnaire was used to collect data. The questions were divided into three sections: first, the checklist of drug delivery methods currently in use; second, a Likert scale of desires and attitudes about five dimensions of service quality including confidence, timeliness, reliability, empathy, and facilities; and third, the demographic information, such as gender, age, marital status, employment status, income per month classified as less than 10,000 THB (less than 263.85 USD), 10,000–20,000 THB (263.85–527.70 USD), 20,001–30,000 THB (527.70–791.56 USD), or more than 30,001 THB (more than 791.56 USD), personal medical care welfare divided into three schemes: (1) Civil Servant Medical Benefit Scheme (CSMBS) which is a fringe benefit to government employees and retirees and their dependents including parents, spouse and not more than three children (less than 20 years old), (2) Social Security Scheme (SSS), which is insurance designed for employees of a private company or non-governmental entity, and (3) Universal Coverage Scheme (UC) which is health insurance for Thai citizens who are not covered by the CSMBS or SSS schemes, distance from home to hospital, underlying disease, the number of medications taken, frequency of medicine refills, and whether any medication delivery method had been used, as illustrated in Supplementary Materials.

The questionnaire was qualified by an expert. An index of item objective congruence (IOC) was used for the content validity test, and Cronbach alpha was conducted for the reliability test. All questions had an IOC of more than 0.6 and an alpha coefficient of more than 0.7.

### 2.4. Data Analysis

Demographic characteristics of all patients were descriptively analyzed and reported as numbers, means, and percentages. A *t*-test was used to compare the two groups’ requirements and attitudes toward medicine delivery services. Additionally, one-way ANOVA was used to compare more than two groups. All of the statistical analyses were performed using IBM SPSS Statistics software version 28.0.0.0 (190), and statistical significance was set at *p* < 0.05.

### 2.5. Ethics Approval

The study was conducted in accordance with the Declaration of Helsinki and approved by the Human Research Ethics Committee of the Walailak University (HREC WU; registration number WUEC-21-002-01, on 6 January 2021).

## 3. Results

Four hundred and six patients were selected to participate in the study; the characteristics of the study’s patients are illustrated in Table 1. The most numerous groups were females (65.8%), ≥60 years old (26.4%), had incomes under 10,000 THB (32.8%), enrolled in the CSMBS, lived within a 15 km distance of the hospital (39.2%), had hypertension (27.6%), and had medicine refills every 3 months (49.3%) with conventional medicine delivery services (85.7%).

### 3.1. The Desire for Medicine Delivery Method

The results of 406 patients’ desire scores, shown in Table 2, found that only the SHPH medicine delivery method received a high level of desire; the rest were scored at medium, as follows: the SHPH (3.38 out of 5), conventional (3.31 out of 5), drug stores (3.12 out of 5), postal pharmacies (3.04 out of 5), and drive-thru (2.21 out of 5) delivery methods, respectively. 

From subgroup analysis with personal characteristics, the results showed that gender determined the difference in the postal pharmacy medicine delivery service desire score (*p*-value = 0.031); income per month determined the difference in SHPH medicine delivery service desire score (*p*-value = 0.001); distance from home to the hospital determined the difference in conventional and SHPH drug delivery service desire score (*p*-value = 0.046 and 0.000, respectively); frequency of medicine refills determined the difference in SHPH and drug store delivery service desire score (*p*-value = 0.004 and 0.000, respectively), and history of medicine delivery service received determined the difference in postal pharmacy medicine delivery service desire score (*p*-value = 0.002).

For the conventional drug delivery method, the distance from home to hospital was a significant factor (*p*-value = 0.046). The distances between 0–15 and 16–30 km were the factors with high level desire scores (3.47 and 3.54 out of 5, respectively). It may be concluded that patients will desire to receive their medicines at the hospital pharmacy department only when the distance between their home and hospital does not exceed 30 km, and the desire level decreases when the distance is longer.

For the drug store delivery method, the frequency of medicine refills was a significant factor (*p*-value = 0.000). Patients who had medicine refills every 1 month highly desired to receive their medicines at drug stores near their homes (3.51 out of 5). On the other hand, there were lower desire scores among patients who had medicine refills every 2 months or more. For the postal pharmacy delivery method, both male and female desire scores were at the medium level (3.24 and 2.94 out of 5, respectively), and patients who had experience with the postal pharmacy delivery method reported desire at a high level (4.75 out of 5), while the rest were given medium desire scores.

Interestingly, the SHPH delivery method affected various factors including income per month, distance from home to hospital, and frequency of medicine refills. Patients who had income of less than 10,000 THB per month reported desire scores on a high level (3.71 out of 5). Patients who lived further than 15 km from the hospital reported desire scores at a high level. This result was related with the factor of conventional drug delivery method desire; it may be concluded that patients whose homes were less than 30 km to the hospital tended to prefer the conventional medicine delivery method, while patients whose homes were more than 30 km from the hospital tended to prefer the SHPH medicine delivery method, as illustrated in Table 2.

### 3.2. Attitudes toward Medicine Delivery Methods

Attitudes toward quality of medicine delivery methods consisted of five dimensions: confidence, timeliness, reliability, empathy, and facilities. The results indicated that NCD patients had positive attitudes toward SHPH and drug store medicine delivery methods that could be seen from a high level of attitude scores across all dimensions, while postal pharmacy and drive-thru delivery methods received a medium level of attitude scores across all dimensions. 

In terms of the confidence dimension, for male patients, the results indicated a significance (*p*-value = 0.034) of drug stores. In terms of timeliness, the results were significant for those with a monthly income of more than 30,000 THB by drive-thru (*p*-value = 0.003), health insurance/UC by SHPH (*p*-value = 0.003), CSMBS by drug stores (*p*-value = 0.008), another medical care scheme by postal pharmacies (*p*-value = 0.044), whose distance from home to hospital was 46–60 km by SHPH (*p*-value = 0.010), who had more than four items of prescribed drugs from drug stores (*p*-value = 0.030), or less than 5 items from postal pharmacies (*p*-value = 0.024), and patients who had ever received drugs from the SHPH (*p*-value = 0.027). Regarding the reliability dimension, for males the results were significant for medicines delivered by SHPH (*p*-value = 0.027) and patients who had ever received drugs from the SHPH (*p*-value = 0.020). In terms of the empathy dimension, the results were significant for those with a monthly income of more than 30,000 THB by drive-thru (0.028), CSMBS by drug stores (*p*-value = 0.034), and patients that had ever received drugs from the SHPH (*p*-value = 0.011). In terms of the physical dimension, the results were significant for those with a monthly income of more than 30,000 THB by drive-thru (*p*-value = 0.004), whose numbers of prescribed drugs were more than 4 items and delivered by drug stores (*p*-value = 0.049), as illustrated in Table 3 and Table 4.

## 4. Discussion

This study highlighted Thai NCD patients’ desires and attitudes regarding five medicine delivery methods including conventional drug delivery, the SHPH, drug stores, postal pharmacies, and drive-thru. The service fee for each medicine delivery scheme depends on medical care schemes. For instance, the CSMBS group does not have out-of-pocket payments because their medical care scheme covers this payment. The SHPH had the medicine delivery services that NCD patients most desired. The drive-thru route was the less-preferred medicine delivery service. It may be reasonable to say that medicine delivery service via drive-thru in Thailand was less significant. The number of patients who experienced using drive-thru medicine delivery services was less. On the other hand, many countries such as the UK, Australia, Taiwan, Malaysia, Croatia, Jordan, and recently, the United Arab Emirates (UAE) and Qatar, have reported drive-thru medicine delivery services since 2008 [11]. Additionally, Thailand’s policy determines that NCD patients are supplied by hospitals. Thus, limitations of medicine delivery services by drive-thru in Thailand may expose the problem of insufficiently staffed pharmacies and incomplete pharmacy workflow patterns. 

According to the conventional medicine delivery scheme and SHPH, patients’ preferences are significantly associated with the distance between the hospital and their homes. The SHPH provides healthcare on the primary care level and has a lesser capacity and facilities in comparison to secondary and tertiary hospitals. It is the decentralization of care and medicine supply points. However, this healthcare model is located in every district, enabling every patient to access it. Patients’ desired distance for the delivery of medicine through the conventional drug delivery model was less than 30 km. In contrast, patients who lived within less than 60 km of the hospital desired to receive medicine from an SHPH. At the same time, the previous study demonstrated that residence within a 1 to 5 km radius from the home to the clinic was related to a higher number of patients willing to pay for delivery service [9]. With this phenomenon, it may be reasonable to hypothesize that patients were confident in the government’s healthcare services. Additionally, the group of patients with a monthly income of less than 10,000 THB preferred to receive drugs from an SHPH, since most patients in this group were under the Universal Coverage Scheme. The SHPH possesses essential national drugs [12] and incurs fewer indirect costs. The income of the patients leads them to select their ideal type of medicine delivery service; for example, the high income group will select other medicine delivery services. Similarly, a previous study demonstrated that higher income patients were more likely to be postal pharmacy users [13]. Moreover, the group that had a frequency of medicine refills of every 3 months preferred to receive drugs from an SHPH. Most patients in this group have stable signs and symptoms. Consequently, they do not need follow-up at a laboratory or require a physical examination by a doctor. While the patients with medicine refills every 1 month demonstrated unstable signs and symptoms as a group, they preferred to receive the drug from a drug store that employs a pharmacist. In addition, medicine delivery services from drug stores use less time in comparison to the SHPH [13,14].

The results indicated that most patients have positive attitudes toward medicine delivery by drug stores in terms of all four dimensions (confidence, time, reliability, and empathy). It may be reasonable to hypothesize that drug stores that were drug receiving channels and employed a pharmacist were available in all areas and had shorter waiting times. In terms of the physical dimension, the results indicated that patients’ attitudes toward medicine delivery service were more positive towards the SHPH. 

Interestingly, in terms of attitudes toward medicine delivery services, the results indicated that those with a monthly income of more than 30,000 THB favored the drive-thru due to the time dimension. Most patients in this study had never received drugs by drive-thru. Thus, they may be aware of the high costs of that channel. A patient who has an income of more than 30,000 THB per month may be more willing to adopt drive-thru delivery due to their experience with general online shopping [14]. In terms of the dimensions of reliability and empathy, most patients relied on government healthcare. Thus, because patients have always received their drugs via government healthcare services, they are experienced the same.

## 5. Conclusions

NCD patients desire for their drugs to be delivered by SHPH medicine delivery services. Thai NCD patients have positive attitudes toward SHPH and drug store medicine delivery methods that can be seen from a high level of attitude scores across all dimensions, while postal pharmacy and drive-thru delivery methods received a medium-level attitude scores across all 5 dimensions.

## Figures and Tables

**Table 1:** Baseline characteristics of NCD patients that were recruited for the analysis (*n* = 406).

Characteristics	Number (%)
Female	267 (65.8)
Age	
18–20 years	15 (3.7)
21–30 years	44 (10.8)
31–40 years	77 (19.0)
41–50 years	82 (20.2)
51–60 years	81 (20.0)
Older than 60 years	107 (26.4)
Income per month (THB)	
Less than 10,000	133 (32.8)
10,000–20,000	128 (31.5)
20,001–30,000	51 (12.6)
More than 30,001	94 (23.2)
Medical care scheme	
CSMBS ^1^	163 (40.2)
Health insurance/UC ^2^	148 (36.5)
Social Security Scheme	73 (18.0)
Distance from home to hospital (km)	
0–15	159 (39.2)
16–30	95 (23.4)
31–45	48 (11.8)
46–60	29 (7.6)
61–75	0
76–90	43 (10.6)
>90	32 (7.9)
Underlying disease	
Hypertension	
Cardiovascular	112 (27.6)
Bone and joint disease	109 (26.9)
Genitourinary disease	50 (12.3)
Diabetes mellitus	49 (12.1)
Gastrointestinal and hepatic disease	47 (11.6)
Otorhinolaryngologic disease	37 (9.1)
Kidney disease	39 (9.6)
Others	41 (10.1)
Number of prescribed drugs	57 (4.0)
1–4 items	257 (63.3)
>4 items	149 (36.7)
Frequency of medicine refills	
Every 1 month	45 (11.1)
Every 2 months	55 (13.5)
Every 3 months	200 (49.3)
Every 4 months	66 (16.3)
More than 4 months	40 (9.9)
History of medicine delivery service	
Conventional drug delivery method	348 (85.7)
Sub-district health promotion hospital	37 (9.1)
Drug stores	13 (3.2)
Postal pharmacy	8 (2.0)
Drive-thru	0

^1^ CSMBS, Civil servant medical benefit scheme. ^2^ UC, Universal Coverage Scheme.

**Table 2:** The personal factors related to the desired medicine delivery methods of NCD patients (*n* = 406).

Personal Factor	Conventional Drug Delivery Model		SHPH		Drug Stores		Postal Pharmacy		Drive-Thru	
	Score	*p*-Value ^†^	Score	*p*-Value ^†^	Score	*p*-Value ^†^	Score	*p*-Value ^†^	Score	*p*-Value ^†^
Total mean	3.31		3.38		3.12		3.04		2.21	
Gender										
Male	3.16	0.169	3.41	0.722	3.15	0.65	3.24	**0.031**	2.12	0.323
Female	3.39		3.36		3.10		2.94		2.26	
Income per month (THB)										
<10,000	3.38	0.358	3.71	**0.001**	3.01	0.542	3.01	0.855	2.05	0.386
10,000–20,000	3.16		3.30		3.16		3.04		2.31	
20,001–30,000	3.14		3.35		3.10		3.20		2.22	
>30,000	3.50		3.03		3.21		3.01		2.31	
Distance from home to the hospital (km)										
0–15	3.47	**0.046**	3.03	**0.000**	3.28	0.219	2.96	0.442	2.27	0.188
16–30	3.54		3.52		2.99		2.89		2.11	
31–45	2.88		3.63		3.13		3.27		2.25	
46–60	2.76		3.93		3.07		3.17		2.17	
61–75 ^1^	-		-		-		-		-	
76–90	3.26		3.74		3.00		3.14		1.88	
>90	3.06		3.38		2.84		3.31		2.69	
Frequency of medicine refills										
1 month	2.96	0.199	3.53	**0.004**	3.51	**0.000**	3.20	0.207	1.96	0.138
2 months	3.69		3.07		2.87		2.87		2.51	
3 months	3.35		3.57		3.05		2.93		2.11	
4 months	3.18		2.97		3.48		3.30		2.33	
>4 months	3.20		3.38		2.73		3.23		2.43	
History of medicine delivery service ^2^										
1	3.33	0.248	3.34	0.056	3.15	0.155	3.01	**0.002**	2.22	0.516
2	3.16		3.76		2.81		3.14		2.16	
3	3.77		3.85		3.38		2.54		1.85	
4	2.38		2.75		2.63		4.75		2.75	
5	3.16		3.76		2.81		3.14		2.16	

Level of desire (mean score): high (3.34–5.00), medium (1.67–3.33), low (0.00–1.66). The bold alphabets are statistical significance result. ^†^ *t*-test analysis was conducted using SPSS, when *p* < 0.05 was considered significant. ^1^ There are no respondents who resided within the distance of 61–75 km from hospital. ^2^ History of medicine delivery service included (1) conventional drug delivery, (2) Sub district health promotion hospital, (3) drug stores, (4) postal pharmacy, and (5) drive-thru.

**Table 3:** The personal factors related to the five attitude scores for medicine delivery methods used by NCD patients (*n* = 406).

Personal Factor	Confidence Dimension ^1^				Reliability Dimension ^1^				Empathy Dimension ^1^			
	1	2	3	4	1	2	3	4	1	2	3	4
Total mean	3.69	3.72	3.28	3.28	3.65	3.72	3.27	3.20	3.64	3.68	3.13	3.04
Gender												
Male	3.71	3.80	3.35	3.28	3.64	3.70	3.33	3.27	3.68	3.74	3.28	3.23
Female	3.70	3.64	3.19	3.20	3.62	3.67	3.15	3.09	3.63	3.63	3.01	3.22
*p*-value	0.193	**0.034**	0.268	0.219	**0.027**	0.053	0.802	0.589	0.591	0.225	0.295	0.92
Income per month (THB)												
<10,000	3.75	3.60	3.2	3.06	3.64	3.59	3.28	3.01	3.60	3.61	3.20	2.88
10,000–20,000	3.73	3.67	3.25	3.24	3.69	3.63	3.17	3.14	3.75	3.57	3.01	2.95
20,001–30,000	3.62	3.86	3.17	3.3	3.73	3.82	3.21	3.31	3.70	3.87	3.01	3.12
>30,000	3.65	3.79	3.33	3.39	3.47	3.81	3.19	3.27	3.53	3.77	3.14	3.26
*p*-value	0.631	0.161	0.648	0.056	0.270	0.229	0.855	0.127	0.261	0.132	0.375	**0.028**
Medical care scheme												
CSMBS	3.66	3.79	3.29	3.29	3.57	3.84	3.18	3.24	3.57	3.82	3.10	3.13
Health insurance/UC	3.79	3.58	3.22	3.14	3.73	3.56	3.31	3.04	3.76	3.51	3.17	2.94
Social Security Scheme	3.68	3.74	3.13	3.19	3.66	3.59	3.02	3.08	3.69	3.66	2.90	2.89
Others	3.49	3.67	3.42	3.50	3.25	3.68	3.52	3.48	3.25	3.59	3.25	3.18
*p*-value	0.226	0.137	0.425	0.243	0.117	0.059	0.112	0.089	0.050	**0.034**	0.223	0.173
Distance from home to hospital (km)												
0–15	3.64	3.72	3.10	3.18	3.55	3.68	3.14	3.10	3.53	3.65	2.98	2.92
16–30	3.66	3.64	3.38	3.30	3.56	3.66	3.16	3.23	3.65	3.68	3.01	3.08
31–45	3.86	3.88	3.32	3.40	3.76	3.81	3.17	3.33	3.79	3.75	3.21	3.28
46–60	3.95	3.55	3.17	2.98	3.88	3.55	3.33	2.97	3.78	3.62	3.26	2.84
76–90	3.78	3.75	3.42	3.19	3.88	3.84	3.58	3.08	3.87	3.83	3.41	3.14
>90	3.56	3.57	3.26	3.22	3.44	3.50	3.23	3.13	3.55	3.39	3.23	2.95
*p*-value	0.141	0.429	0.131	0.456	0.097	0.589	0.217	0.522	0.177	0.460	0.090	0.218
Number of prescribed drug												
1–4 items	3.65	3.64	3.22	3.17	3.57	3.63	3.18	3.10	3.60	3.60	3.10	2.99
>4 items	3.79	3.80	3.28	3.33	3.72	3.78	3.28	3.24	3.72	3.78	3.11	3.08
*p*-value	0.784	0.765	0.402	0.585	0.832	0.568	0.368	0.788	0.164	0.065	0.919	0.381
History of medicine delivery service ^2^												
1	3.62	3.91	3.47	3.38	3.6	3.69	3.21	3.16	3.64	3.67	3.11	3.01
2	3.93	3.68	3.1	3.08	4.03	3.58	3.12	3.03	3.96	3.70	2.88	3.05
3	3.68	3.69	3.24	3.24	3.31	3.81	3.38	3.19	3.04	3.69	3.31	3.23
4	3.65	3.69	3.81	3.10	3.25	3.50	3.81	3.25	3.38	3.50	3.56	3.19
*p*-value	0.272	0.819	0.167	0.676	**0.020**	0.813	0.336	0.862	**0.011**	0.955	0.220	0.815

Level of attitude (mean score): high (3.34–5.00), medium (1.67–3.33), low (0.00–1.66). The bold alphabets are statistical significance result. ^1^ The medicine delivery methods included (1) sub district health promotion hospital, (2) drug stores, (3) postal pharmacy, and (4) drive-thru. ^2^ History of medicine delivery service included (1) conventional drug delivery, (2) sub district health promotion hospital, (3) drug stores, and (4) postal pharmacy.

**Table 4:** The personal factors related to the five attitude scores for medicine delivery methods used by NCD patients (*n* = 406).

Personal Factor	Time Dimension ^1^				Physical Dimension ^1^			
	1	2	3	4	1	2	3	4
Total mean	3.65	3.68	3.22	3.24	3.57	3.46	3.09	2.96
Gender								
Male	3.63	3.74	3.25	3.23	3.62	3.41	3.20	2.95
Female	3.67	3.16	3.14	3.22	3.49	3.47	2.96	2.97
*p*-value	0.712	0.225	0.295	0.920	0.366	0.054	0.508	0.227
Income per month (THB)								
<10,000	3.73	3.53	3.21	2.94	3.54	3.36	2.97	2.72
10,000–20,000	3.71	3.70	3.11	3.30	3.52	3.45	2.96	2.98
20,001–30,000	3.65	3.63	3.25	3.37	3.51	3.56	3.05	3.09
>30,000	3.47	3.79	3.20	3.41	3.54	3.52	3.24	3.21
*p*-value	0.183	0.269	0.787	**0.003**	0.996	0.527	0.155	**0.004**
Medical care scheme								
CSMBS	3.53	3.85	3.22	3.32	3.44	3.55	3.10	3.08
Health insurance/UC	3.84	3.48	3.22	3.07	3.67	3.36	3.05	2.88
Social Security Scheme	3.68	3.66	2.92	3.17	3.52	3.43	2.88	2.82
Others	3.20	3.41	3.50	3.59	3.32	3.34	3.11	3.11
*p*-value	**0.003**	**0.008**	**0.044**	0.071	0.116	0.345	0.475	0.168
Distance from home to hospital (km)								
0–15	3.45	3.70	3.04	3.22	3.47	3.49	2.96	2.97
16–30	3.71	3.64	3.22	3.32	3.53	3.42	2.96	2.97
31–45	3.78	3.79	3.29	3.42	3.65	3.54	3.03	3.11
46–60	4.00	3.48	3.21	2.74	3.76	3.38	3.28	2.74
76–90	3.91	3.83	3.45	3.06	3.65	3.51	3.33	3.1
>90	3.66	3.20	3.20	3.28	3.33	3.19	3.11	2.66
*p*-value	**0.010**	0.074	0.207	0.094	0.396	0.607	0.21	0.310
Number of prescribed drugs								
1–4 items	3.61	3.58	3.10	3.14	3.54	3.39	3.00	2.89
>4 items	3.73	3.80	3.33	3.35	3.52	3.55	3.11	3.09
*p*-value	0.237	**0.030**	**0.024**	0.063	0.693	**0.049**	0.243	0.799
History of medicine delivery service ^2^								
1	3.63	3.64	3.18	3.23	3.51	3.45	3.02	2.95
2	4.07	3.72	3.03	3.28	3.88	3.49	3.07	3.05
3	3.38	3.85	3.27	2.77	3.19	3.46	3.23	3.23
4	3.31	3.88	4.00	3.06	3.25	3.31	3.56	2.81
*p*-value	**0.027**	0.782	0.085	0.448	0.060	0.975	0.403	0.702

Level of attitude (mean score): high (3.34–5.00), medium (1.67–3.33), low (0.00–1.66). The bold alphabets are statistical significance result. ^1^ The medicine delivery methods included (1) sub district health promotion hospital, (2) drug stores, (3) postal pharmacy, and (4) drive-thru. ^2^ History of medicine delivery service included (1) conventional drug delivery, (2) sub district health promotion hospital, (3) drug stores, and (4) postal pharmacy.

## Supplementary Materials

The following supporting information can be downloaded at: https://www.mdpi.com/article/10.3390/ijerph192013571/s1, Questionnaire for Collecting Data in This Study.
