# Supplementary material for: Desires and Attitudes towards Telepharmacy Medicine Delivery
Source: Int J Environ Res Public Health. 2022 Oct 20;19(20):13571. doi: 10.3390/ijerph192013571 (PMC9603625; doi:10.3390/ijerph192013571)
Supplement: Supplementary file 1 [file ijerph-19-13571-s001.zip › ijerph-1953437-supplementary.pdf]

Supplementary Materials

S1. Questionnaire for Collecting Data in This Study

Section 1: The score of the desires towards medicine delivery method. Please √ in the table for score of the desires towards medicine delivery method.

5 = Strongly need or agree 4 =Need or Agree 3 = Neutral 2 = No need or Disagree 1 = Strongly no need or disagree

The Medicine Delivery Method	The Score of the Desires towards Medicine Delivery Method				
	5	4	3	2	1
1. Conventional method					
2. Subdistrict health promotion hospital					
3. Drug stores					
4. Postal pharmacy					
5. Drive-thru					

Section 2: The questions for desires and attitudes towards medicine delivery method. Please fill the score in the table.

5 = Strongly need or agree 4 =Need or Agree 3 = Neutral 2 = No need or Disagree 1 = Strongly no need or disagree

Questions	The Score of the Medicine Delivery Method				
	Conventional Method	Subdistrict Health Promotion Hospital	Drug Stores	Postal Pharmacy	Drive-Thru
1. How many scores for drug quality do you get in the confidence dimension?					
2. How many scores for drug accuracy do you get in the confidence dimension?					
3. How many scores for drug counseling do you get in the confidence dimension?					
4. How many scores for drug solving of pharmacists (i.e., adverse drug reaction) do you get in the confidence dimension?					
5. How many scores for remote communication of pharmacists do you get in the confidence dimension?					
6. How many scores for personal information concealment of pharmacists do you get in the confidence dimension?					
7. How many scores for transportation do you take in the time dimension?					
8. How many scores for dispensing process do you get in the time dimension?					
9. How many scores for dispensing process do you get in the reliability dimension?					
10. How many scores for pharmacists who dispensed medicine to you are in the reliability dimension?					

11. How many scores for pharmacists who dispensed medicine to you are in the empathy dimension?	
12. How many scores for pharmacist's personality who dispensed medicine to you are in the empathy dimension?	
13. How many scores for satisfaction to payment that doesn't cover the medical care scheme?	
14. How many scores for physical appearance to the dispensary?	
15. How many desire scores to receive medicine delivery service?	

Section 3: Please answer your demographic information in the space.

Gender: Age: Marital status: Employment status:

The income per month (1 US dollar equals 37.9 THB):

- ☐ Less than 10,000 THB
- ☐ 10,000–20,000 THB
- ☐ 20,001–30,000 THB
- ☐ more than 30,001 THB

Medical care scheme:

- ☐ Civil servant medical benefit scheme
- ☐ Health insurance/Universal Coverage Scheme
- ☐ Social Security Scheme

Distance from home to hospital (km): Underlying diseases:

The number of medications taken (items): Frequency of medicine refills:

Previous medication delivery services had been used:

- ☐ Conventional drug delivery model
- ☐ Subdistrict health promotion hospital
- ☐ Drug stores
- ☐ Postal pharmacy
- ☐ Drive-thru
